# The first detection of swine orthopneumovirus in a pig farm in Sweden: a case report

**DOI:** 10.1186/s40813-025-00473-2

**Published:** 2025-11-05

**Authors:** Eveliina Breukers, Hyeyoung Kim, Fereshteh Banihashem, Kristofer Andersson, Mikael Leijon, Rebecka Westin, Marie Sjölund, Siamak Zohari

**Affiliations:** 1 Department of Clinical Sciences, Swedish University of Agricultural Sciences, Uppsala, SE-75007 Sweden; 2 Deparment of Animal Health and Antimicrobial Strategies, Swedish Veterinary Agency, Uppsala, SE-75189 Sweden; 3 Department of Epidemiology, Surveillance and Risk Assessment, Swedish Veterinary Agency, Uppsala, SE-751 89 Sweden; 4 Department of Microbiology, Swedish Veterinary Agency, Uppsala, SE-751 89 Sweden; 5 Department of Applied Animal Science and Welfare, Swedish University of Agricultural Sciences, Skara, SE-532 23 Sweden; 6 Farm and Animal Health (Gård&Djurhälsan), Skara, SE-532 40 Sweden

**Keywords:** Metagenomic analysis, Orthopneumovirus, Polymicrobial respiratory infection, Respiratory virus, Swine

## Abstract

**Background:**

Respiratory diseases are globally a major challenge in today’s pig production. Despite the efforts to manage the disease, the number of pigs affected is still increasing, indicating gaps in the current knowledge. In 2016, a novel pneumovirus, swine orthopneumovirus, was detected in the USA. Since then, the virus has been detected in a few European countries and in South Korea. However, the wider distribution of the virus is still greatly unknown, as well as its clinical relevance.

**Case presentation:**

This report describes the first detection of swine orthopneumovirus in a Swedish pig herd. The virus was detected as the result of an investigation conducted between September 2023 to June 2024, where all clinical samples (*n* = 682) sent to the Swedish Veterinary Agency for diagnostic purposes from 112 Swedish pig farms exhibiting clinical signs of respiratory disease were screened for the presence of swine orthopneumovirus. The virus was detected in one piglet producing farm that had a respiratory disease outbreak in autumn 2023, which presented with cough and nasal discharge. In November 2023, 11 nasal swabs were collected, of which 9 were PCR-positive for swine orthopneumovirus. In addition, each sample was also PCR-positive for *Mesomycoplasma hyorhinis* and *Pasteurella multocida*, and 2 samples were PCR-positive for *Actinobacillus pleuropneumoniae*, indicating a polymicrobial respiratory infection.

**Conclusions:**

This report emphasises the importance of ongoing efforts to identify emerging pathogens and determine their clinical significance. Therefore, further research is needed to assess the distribution and potential clinical relevance of swine orthopneumovirus.

**Supplementary Information:**

The online version contains supplementary material available at 10.1186/s40813-025-00473-2.

## Background

Respiratory diseases are globally one of the most significant contributors to decreased animal welfare and productivity in pig production, increasing the need for antibiotic treatments and subsequently the risk for the development of antibiotic resistance [1, 2]. Polymicrobial respiratory diseases in pigs are commonly referred to as the porcine respiratory disease complex (PRDC), which is influenced by infectious, management, and environmental factors. Multiple bacteria and viruses have been recognised to play a role in PRDC, but the interaction of the pathogens is not fully understood [3, 4]. In addition, new porcine viruses have emerged but the distribution and clinical relevance of these are not well described [5, 6].

For the last decade, Sweden has produced around 2.5 million slaughter pigs each year [7]. Since 2000, the number of pig producers in Sweden has decreased by 80% while the average herd size has increased which is consistent with international trends. In 2023, there were 1160 registered pig producers with 1.3 million pigs, representing approximately 1% of the pig population in the EU [8, 9]. Sweden has high standards for animal welfare such as strict requirements for the minimum space allowance and all animals must be kept in loose housing at every production stage [10, 11]. In addition, some common porcine pathogens are absent in the Swedish pig population, such as the porcine reproductive and respiratory disease syndrome virus (PRRSV) and Aujeszky’s disease virus. Despite this, the prevalence of respiratory diseases in Swedish pig herds has increased over the last several decades [12] and as many as 50% of pigs in some slaughter batches have signs of pleuritis at meat inspection. Respiratory diseases are also common in other parts of Europe where the prevalence of pleuritis detected during meat inspection varies between 6 and 50% [13].

In 1998, serological evidence of pneumovirus infection in pigs in Northern Ireland was described because of apparent cross-reaction of pig sera with bovine respiratory syncytial virus (BRSV) antigen [14]. In 2016, a novel porcine respiratory virus, swine orthopneumovirus (SOV) of the genus *Orthopneumovirus*, was discovered and sequenced in the USA [6]. It has since been detected in Spain [15], Germany [16], and South Korea [17]. Serological evidence of SOV has been reported in France [18]. Previously, there was no evidence of the presence of SOV in the Nordic countries. However, very few veterinary laboratories in Europe test for SOV on a routine basis, resulting in a lack of knowledge of its distribution or how much it contributes to the development of PRDC. Several recent reports have indicated the presence of the virus in various European countries. We have investigated the presence of SOV in Swedish pigs by screening incoming samples submitted for routine diagnostics for the virus. The aims of this report are to present the first detection of SOV in Sweden and to describe the genetic diversity of SOV strains that circulate in Europe.

## Screening survey

**Fig. 1:**
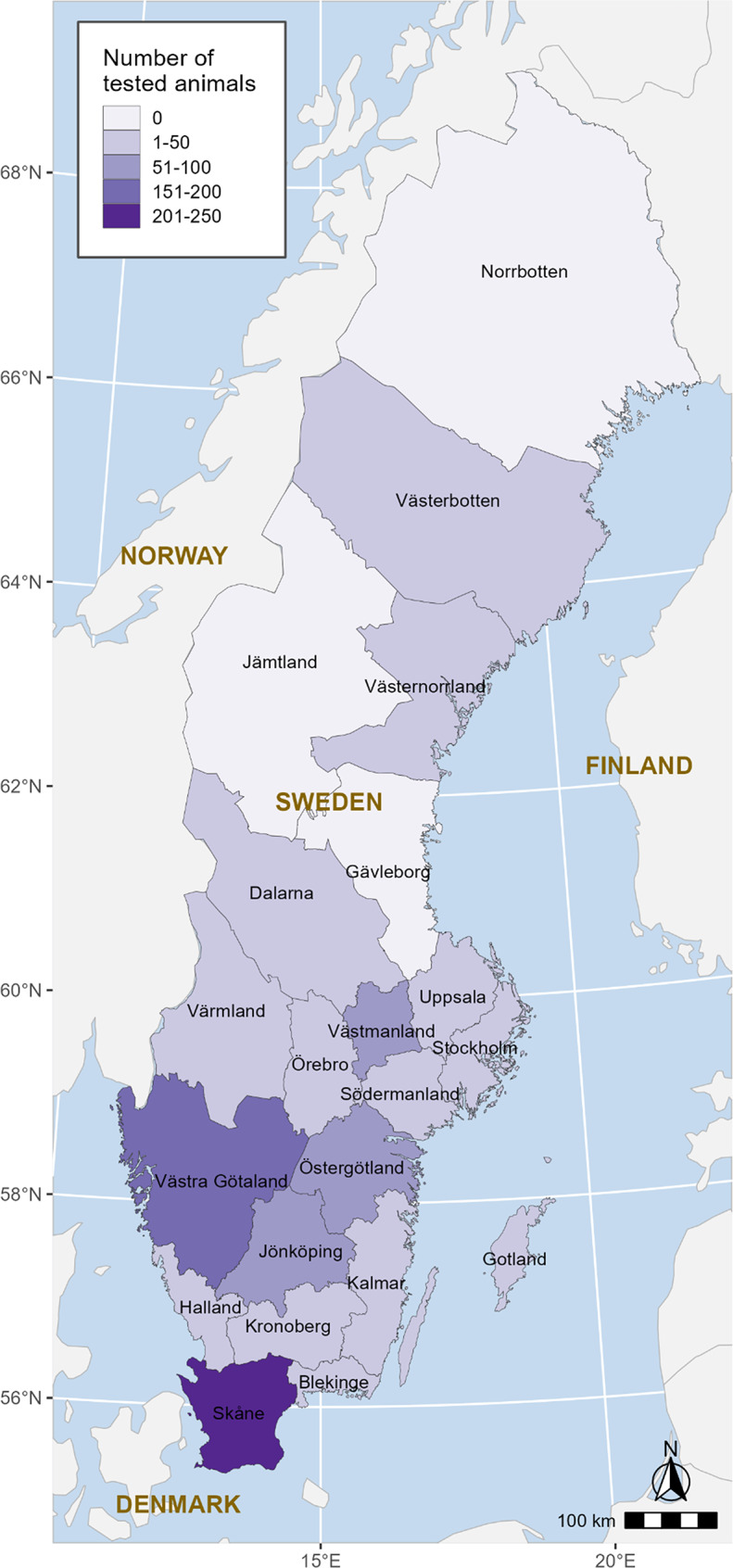
The geographic distribution of the tested animals screened for swine orthopneumovirus by a real-time reverse transcription quantitative PCR

As part of an ongoing screening project between September 2023 and June 2024, all clinical samples (*n* = 682) submitted to the Swedish Veterinary Agency (SVA) for diagnostics as a part of routine veterinary care from Swedish pig farms exhibiting clinical signs of respiratory disease (*n* = 112), were screened for the presence of SOV using previously described protocol [16]. The screening project aimed to survey the occurrence and incidence of infections in connection with SOV and determine if it is a potential contributor to the PRDC in the Swedish pig population. The distribution of tested animals represents the geographical distribution of the pig population in Sweden (Fig. 1). The visualization of the maps was performed using R version 4.4.1 [19].

## Case presentation

Swine orthopneumovirus was detected from 1 out of 112 farms investigated, where 9 out of 11 samples were positive in pigs 3–5 weeks of age. In autumn 2023, this piglet producing farm with 1100 sows in a weekly batch farrowing system suffered from a respiratory disease outbreak. In October 2023, coughing had occurred in grower pigs in three consecutive batches shortly after weaning. In November, the herd veterinarian was called out since now also three-week-old piglets in one farrowing unit had started to cough. During the visit, pigs presented with cough and nasal discharge were detected in four different units, hereafter referred to as: units A-D. A high number of pigs were coughing in unit B, C and D (three to five-week-old pigs), spread throughout the units. In unit A (two-weeks-old piglets), only a few piglets were affected. The herd veterinarian collected in total 11 nasal swab samples from these four units. Sampling was done from piglets showing clear signs of disease, such as cough and nasal discharge, resulting in one sample (sample no. 1) from unit A, four samples (sample no. 2–5) from unit B from three-week-old piglets, three samples (sample no. 6–8) from unit C from recently weaned four-week-old pigs, and three samples (sample no. 9–11) from unit D from five-week-old pigs.

According to the farm’s standard management routines, piglets were vaccinated against porcine circovirus type 2 (PCV2) and *Mesomycoplasma hyopneumoniae* (Mhyo) with Porcilis PCV Mhyo injectable vaccine at three weeks of age. No medical treatments for pneumonia were performed in the farrowing units, but the number of individuals treated with injectable antibiotics (Procaine benzylpenicillin) for acute pneumonia in the grower units increased during the outbreak. On average, 4.6% of the piglets were individually treated in the four sampled batches (variation 3.4–5.8%) compared to 2.8% in the eight batches preceding the outbreak (variation 1.6–4.2%). No flock treatments were performed in the farrowing units, nor in the growing units. Mortality in the grower units after weaning until at 10 weeks of age in the four sampled batches (including euthanised pigs) varied between 1.9 and 3.7% compared to 1.6–3.2% in the eight batches weaned before the outbreak started.

The samples were submitted to the SVA for diagnostic purposes. They were initially tested for several respiratory pathogens included in the routine diagnostic workflow. The respiratory diagnostic panel includes a real-time reverse transcription quantitative PCR (RT-qPCR) for *Actinobacillus pleuropneumoniae* (APP), *Pasteurella multocida* (Pm), Mhyo, *Mesomycoplasma hyorhinis* (Mhr), and swine influenza A virus (swIAV). At the time of sampling, the main interest was to know if influenza virus was present at the farm as human influenza virus had been detected there previously. For this reason, some porcine pathogens, such as porcine reproductive and respiratory syndrome virus (PRRSV), were not included in the diagnostic panel. In addition, the Swedish pig population is free from PRRSV and has a national monitoring program for PRRS to ensure the freedom from the disease. The decision to analyse samples for PRRSV at a suspicion of disease is made by the Swedish Board of Agriculture based on the risk assessment provided by SVA. As the samples from the farm were collected at the time of respiratory disease outbreak, all samples were included in the SOV screening.

## Detection of SOV

After performing RNA extraction (Supplementary methods), samples were analysed for the presence of SOV RNA and were screened with an initial qPCR targeting the N-gene. Positive samples were confirmed using a second qPCR assay targeting the G-gene of SOV [16].

**Fig. 2:**
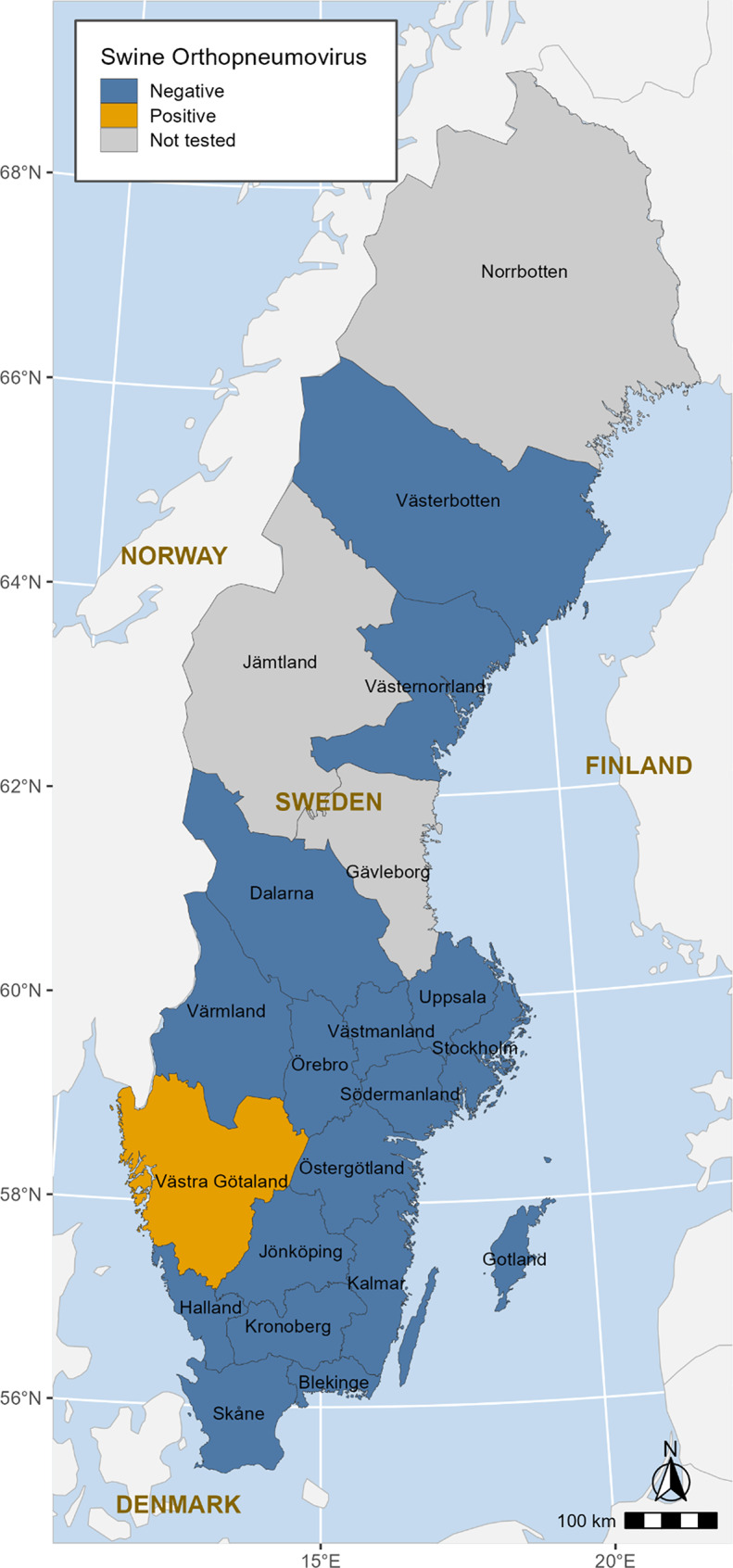
The location of the swine orthopneumovirus positive farm, detected during the screening of clinical samples (*n* = 682) from Swedish pig farms (*n* = 112) exhibiting clinical signs of respiratory disease

All four samples from unit B were positive for SOV, as well as three samples from unit C and two from unit D (Table 1). The farm was the only SOV-positive farm detected during the screening period (Fig. 2). If at least one farm was positive, the area was considered positive. All samples were also PCR-positive for Pm and Mhr, while negative for Mhyo and swIAV. Two samples from unit D were positive for APP. Two SOV-positive samples with the highest viral load (Ct values 17 and 19) were selected for metagenomic next-generation sequencing (mNGS) using the previously described protocol [20].

**Table 1:** Detection of swine respiratory pathogens in a Swedish 1100 Sow piglet producing herd with a RT-qPCR

	Pig age	Sample no.	RT-qPCR (Ct-value)					
			SOV	APP	Pm	Mhyo	Mhr	swIAV
Unit A	2 weeks	1	-	-	+(31.95)	-	+(38.06)	-
Unit B	3 weeks	2	+(27.94)	-	+(25.70)	-	+(23.10)	-
		3	+(19.29)	-	+(26.45)	-	+(25.87)	-
		4	+(17.02)	-	+(26.61)	-	+(23.43)	-
		5	+(19.94)	-	+(21.07)	-	+(21.98)	-
Unit C	4 weeks	6	+(29.23)	-	+(36.40)	-	+(30.78)	-
		7	+(29.42)	-	+(31.95)	-	+(34.27)	-
		8	+(31.97)	-	+(31.88)	-	+(36.79)	-
Unit D	5 weeks	9	+(29.24)	+(35.05)	+(32.44)	-	+(30.21)	-
		10	+(29.16)	+(34.1)	+(31.68)	-	+(29.77)	-
		11	-	-	+(31.86)	-	+(24.59)	-

The nucleotide sequence was compared to the NCBI GenBank database using the BLAST algorithm Search Tool (BLASTn). The amino acid sequences of the putative proteins were compared to proteins in the NCBI GenBank database using the Basic Local Alignment (BLASTp). The complete genomes were generated and uploaded to Genbank (Genbank PP336899-PP336900).

De novo assembly of the SOV-positive samples generated sequences of 14 891 and 14 897 nucleotide base pairs, respectively, and confirmed the presence of 11 open reading frames (NS1, NS2, N, P, M, SH, G, F, M2-1, M2,2 and L) of SOV. The generated whole genome sequences were 93% identical to the KSOV-2202 strain (Table 2), isolated from diseased pigs at commercial farms in South Korea in 2022 [17]. Pairwise amino acid alignments and the comparison of the amino acid sequence of the respective gene between the Swedish SOV and KSOV-2202 revealed the overall amino acid similarity between 90.6% (G-gene) and 98.98% (N-gene). The G protein, which interacts with host cell factors and mediates virus entry, exhibits the highest number of amino acid substitutions (*n* = 39) compared to KSOV-2202. The functional significance of these substitutions has not been explored but changes, particularly in surface-exposed loop regions and potential glycosylation sites could alter viral antigenicity, receptor interactions and immune evasion [21]. The F protein of Swedish SOV exhibits multiple substitutions (*n* = 15) compared to KSOV-2202, particularly in the signal peptide region at the N-terminus (positions 1–20), which may affect how the F protein is processed for folding and maturation inside the infected cells [22, 23].

**Table 2:** Open reading frame (ORF) composition and length and pairwise amino acid identity between SOV ORFs of the Swedish virus and KSOV-2202 are shown here

	Swedish SOV		KSOV-2202 OR701948.1	
ORF	Amino Acids (AA)	AA	Number of AA differences	Identity to S-SOV%
NS1	114	114	9	92,11
NS2	157	157	9	94,27
N	393	393	4	98,98
P	295	295	10	96,62
M	257	257	5	98,06
SH	92	92	6	93,55
G	414	414	39	90,6
F	537	537	15	97,21
M2-1	176	176	3	98,31
M2-2	98	98	1	97,98
L	2038	2038	45	97,7

**Fig. 3:**
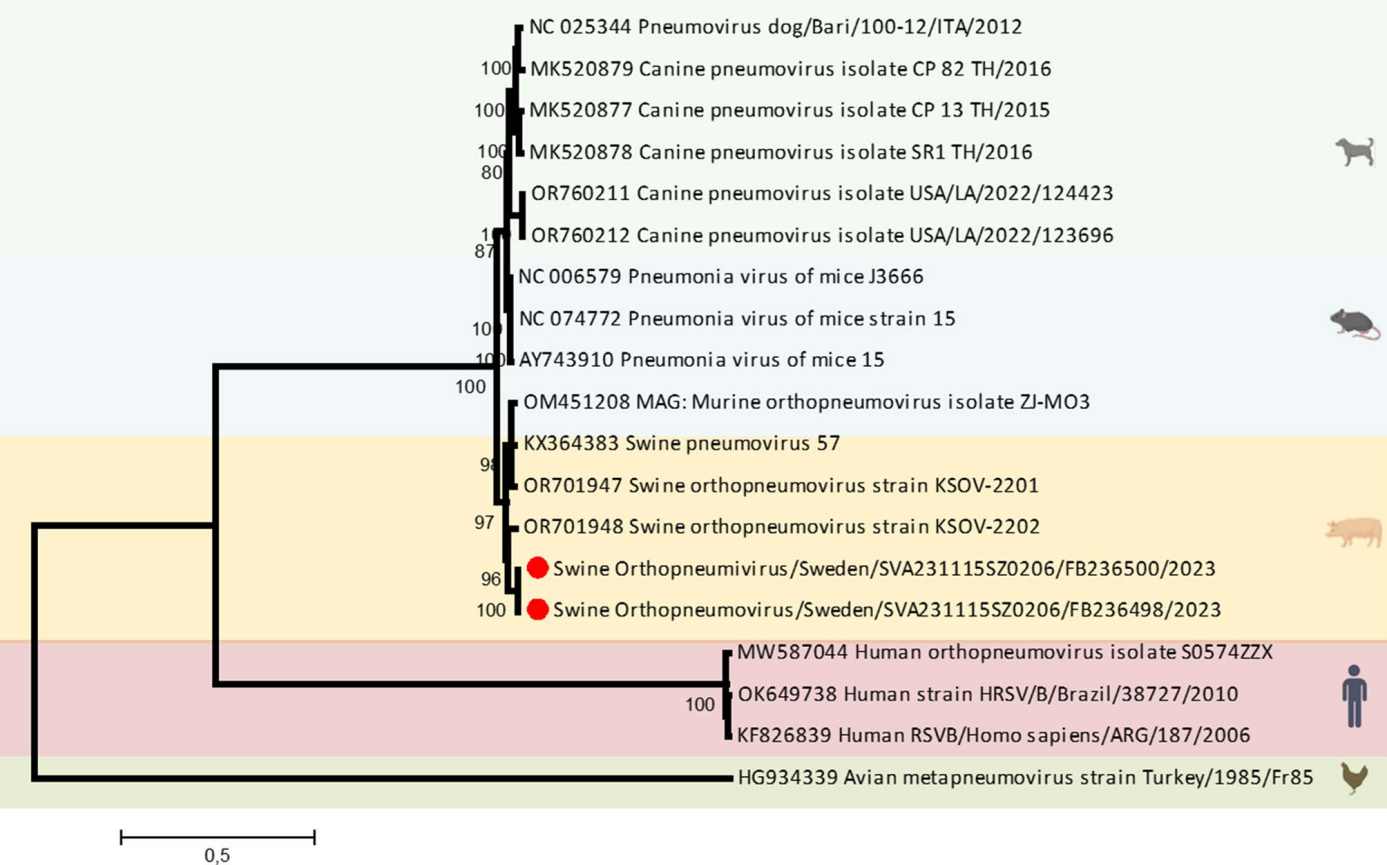
A phylogenetic tree constructed using the complete genomes available for *orthopneumoviruses*. Two swine orthopneumoviruses detected in the case farm are indicated with red dots

To further investigate the genetic relationship of the viruses detected in Sweden, we retrieved all available whole genome sequences of *Orthopneumovirus* from the NCBI GenBank. Seventeen available sequences were aligned, and a phylogenetic tree (Fig. 3.) was constructed using the maximum likelihood method based on the general time reversible model. The percentages of replicate trees in which the associated taxa clustered together in the bootstrap test (2000 replicates) are shown below the branches [24]. Evolutionary analyses were conducted in MEGA7 [25]. Phylogenetic analysis of the Swedish SOV’s whole genome sequence and the F and G gene sequences separately, showed that the Swedish SOV clustered with KSOV-2202 in the same clade as other available swine *Orthopneumoviruses.*

## Discussion and conclusions

To the authors’ knowledge, this is the first time SOV has been detected in pigs in Sweden. Swine orthopneumovirus was detected from 1 out of 112 farms investigated, where 9 out of 11 samples were positive in pigs 3–5 weeks of age. At the time of sampling, the SOV-positive farm was also PCR-positive for APP, Pm, and Mhr, whereas it was negative for Mhyo and swIAV. These findings indicate a polymicrobial respiratory infection. Identifying which pathogen combination, together with environmental and management factors, contributed to the clinical signs is challenging.

Polymicrobial respiratory infection has been shown in other cases of SOV in Spain and South Korea [15, 17]. In Spain, 31 virus combinations were detected on 55 farms with respiratory disease and SOV was detected in 17 farms. The presence of SOV correlated positively with presence of swIAV, porcine respiratory coronavirus (PRCV) and PCV2 in the Spanish herds [15]. In South Korea, some of the SOV-positive samples were also positive for PRRSV-1, PRRSV-2, PCV2, Mhyo, and porcine respirovirus 1 (PRV-1) [17], while swIAV was not present. Sweden is officially free of PRRSV and the freedom of the disease is monitored by both passive and active surveillance [26]. Hence, PRRSV is not thought to contribute to PRDC in Sweden [27].There are no recent studies of the prevalence of PRCV or PCV2 in the Swedish pig herds. However, in general, PRCV has been thought to be present in most herds, usually resulting in subclinical and self-limiting disease [28]. Most pigs are vaccinated against PCV2 because it is required when pigs are sold from piglet-producing herds to specialised fattening herds [29]. The apparent contribution of Mhyo to pneumonia in Swedish herds has decreased since the implementation of age-segregated rearing systems in Sweden in the 1990s [12]. In addition, nucleus herds must be vaccinated against Mhyo [29]. At present, the presence of PRV-1 in Swedish pig herds is not known.

There is no information on how SOV could have been introduced to the farm. As SOV has been relatively recently discovered and not routinely looked for, its clinical contribution remains unknown [16], as well as its potential pathogenicity and transmission routes. In humans and other species, metapneumoviruses are known to cause clinical disease and are transmitted via respiratory secretions [30]. In humans, human respiratory syncytial virus (HRSV), genus *Orthopneumovirus*, leads to respiratory infections, most commonly in infants but also in elderly and immunocompromised patients [31]. Human metapneumovirus, genus *Metapneumovirus*, causes a similar clinical picture to HRSV and is closely related to avian metapneumovirus. Avian metapneumovirus, genus *Metapneumovirus*, causes severe respiratory infection in turkeys and usually results in lethal upper respiratory infection. Bovine respiratory syncytial virus, genus *Orthopneumovirus*, induces clinical disease and can contribute to polymicrobial respiratory infection in cattle. Both BRSV and HRSV transmissions are believed to occur via respiratory secretions. For HRSV, droplet transmission is also believed to occur [30]. If SOV behaves the same way as HRSV or BRSV, transmission via respiratory secretions could be thought to occur, but more research should be done to confirm this. In addition, the possibility of indirect transmission for example via visitors or vehicles, cannot be excluded. At the same time, the transmission of routes of respiratory viruses in general is quite poorly understood, while better tools to detect air-borne viruses are being developed [32]. As the SOV-positive farm did not buy any animals but raised their own animals for breeding and since no animals had been introduced to the farm since 2020, the risk of a recent external introduction of the virus is considered low or negligible. The virus may have been present in the herd for some time but gone unnoticed as it was only recently detected and has not been routinely looked for. As the youngest sampled animal (sample no. 1) was negative, it may be that it either had not been exposed to the virus or the sampling strategy was not able to detect the virus in the unit. If the unit A truly was negative, it would mean that older animals would have been recently exposed to the virus, thus suggesting a recent virus introduction. Whether the virus was already circulating or recently introduced, we want to emphasise the importance of continuous efforts to search for emerging pathogens.

The limitations of the traditional diagnostic methods, such as the inability to detect novel pathogens, can result in delayed or inaccurate diagnosis [33]. On the other hand, mNGS is an unbiased and comprehensive tool for identifying and characterising microorganisms [33, 34]. In addition, over time, the costs of mNGS have reduced [34], making it more accessible. Due to these factors, mNGS is a promising tool for the detection of microorganisms associated with PRDC. However, the presence of a microorganism is not equivalent to a clinically relevant pathogen, like in the case of SOV, whose clinical contribution is still unknown.

This report presents the first detection of SOV in a Swedish pig herd, during a clinical respiratory outbreak, including the presence of other porcine respiratory pathogens. This is also the first time in Europe and the fourth time globally that the whole genome sequence of SOV has been described. This report highlights the importance of continuous efforts to search for emerging pathogens with modern tools such as sequencing, as it can potentially fill some of the knowledge gaps regarding PRDC. Before including SOV in the routine diagnostic panel, its clinical relevance should be defined. To achieve this, experimental studies are often necessary to confirm the causality between the agent and clinical disease. Further studies are also needed to assess the distribution of SOV, to get a better understanding of how widespread the potential impact of SOV is.

## Supplementary Information

Below is the link to the electronic supplementary material.

Supplementary Material 1

## Data Availability

The datasets used and/or analysed during the current study are available from the corresponding author on reasonable request. The generated sequence data are available at www.ncbi.nlm.nih.gov/genbank on the accession number PP336899-PP336900.

## Declarations

### Prior publication

These results have been presented at the European Symposium of Porcine Health Management (ESPHM) 2025 in the form of an oral presentation.

## Abbreviations

APP: Actinobacillus pleuropneumoniae
BRSV: Bovine respiratory syncytial virus
HRSV: Human respiratory syncytial virus
Mhyo: Mesomycoplasma hyopneumoniae
mNGS: Metagenomic next-generation sequencing
Mrh: Mesomycoplasma hyorhinis
PCV2: Porcine circovirus type 2
Pm: Pasteurella multocida
PRDC: Porcine respiratory disease complex
PRV-1: Porcine respirovirus 1
RT-qPRC: Real-time reverse transcription quantitative PCR
SOV: Swine orthopneumovirus
SVA: Swedish Veterinary Agency

## References

[1] Nathues H, Alarcon P, Rushton J, Jolie R, Fiebig K, Jimenez M, Geurts V, Nathues C. Cost of Porcine reproductive and respiratory syndrome virus at individual farm level – An economic disease model. Prev Vet Med. 2017. 10.1016/j.prevetmed.2017.04.006. 28606362 10.1016/j.prevetmed.2017.04.006

[2] Wallgren P, Verdier K, Sjölund M, Zoric M, Hulten C, Ernholm L, Persson Waller K. Jan. Hur mycket kostar sjukdomar för lantbrukets djur? https://www.sva.se/media/8da84dbf7459112/rapport_sjukdomskostnader-lantbruket-del-1_2012.pdf. Accessed 20 2025.

[3] Opriessnig T, Giménez-Lirola LG, Halbur PG. Polymicrobial respiratory disease in pigs. Anim Health Res Rev. 2011;12(2):133–48. 10.1017/S1466252311000120. 22152290 10.1017/S1466252311000120

[4] Thacker EL. Immunology of the Porcine respiratory disease complex. Vet Clin North Am Food Anim Pract. 2001;17(3):551–65. 10.1016/s0749-0720(15)30006-2. 11692508 10.1016/S0749-0720(15)30006-2 PMC7134923

[5] Lau SKP, Woo PCY, Wu Y, Wong AYP, Wong BHL, Lau CCY, Fan RYY, Cai JP, Tsoi HW, Chan KH, Yuen KY. Identification and characterization of a novel paramyxovirus, Porcine parainfluenza virus 1, from deceased pigs. J Gen Virol. 2013;94(Pt 10):2184–90. 10.1099/vir.0.052985-0. 23918408 10.1099/vir.0.052985-0

[6] Hause BM, Padmanabhan A, Pedersen K, Gidlewski T. Feral swine Virome is dominated by single-stranded DNA viruses and contains a novel orthopneumovirus which circulates both in feral and domestic swine. J Gen Virol. 2016;97(9):2090–5. 10.1099/jgv.0.000554. 27417702 10.1099/jgv.0.000554

[7] Swedish Board of Agriculture. Jordbruksverkets statistikdatabas. https://statistik.sjv.se/PXWeb/pxweb/sv/Jordbruksverkets%20statistikdatabas/?rxid=5adf4929-f548-4f27-9bc9-78e127837625. Accessed 17 Jul 2024.

[8] Swedish Board of Agriculture. Lantbrukets djur i juni 2023 Slutlig statistik. 2024. https://jordbruksverket.se/om-jordbruksverket/jordbruksverkets-officiella-statistik/jordbruksverkets-statistikrapporter/statistik/2024-01-31-lantbrukets-djur-i-juni-2023-slutlig-statistik#:~:text=Denna%20statistikrapport%20redovisar%20uppgifter%20om%20antalet%20husdjur%20och,p%C3%A5%20jordbruksf%C3%B6retagets%20storlek%20m%C3%A4tt%20efter%20%C3%A5ker-%20eller%20jordbruksmark. Accessed 17 Apr 2025.

[9] Eurostat. Agricultural production-livestock and meat. 2024. https://ec.europa.eu/eurostat/statistics-explained/index.php?oldid=427096. Accessed 17 Apr 2025.

[10] Animal Protection Index. https://api.worldanimalprotection.org/. Accessed 31 Mar 2025.

[11] SJVFS. 2019:20. https://lagen.nu/sjvfs/2019:20. Accessed 31 Mar 2025.

[12] Wallgren P, Nörregård E, Molander B, Persson M, Ehlorsson CJ. Serological patterns of Actinobacillus pleuropneumoniae, Mycoplasma hyopneumoniae, pasteurella multocida and Streptococcus suis in pig herds affected by pleuritis. Acta Vet Scand. 2016;58(1):71. 10.1186/s13028-016-0252-1. 27716292 10.1186/s13028-016-0252-1 PMC5050615

[13] Maes D, Sibila M, Pieters M, Haesebrouck F, Segalés J, de Oliveira LG. Review on the methodology to assess respiratory tract lesions in pigs and their production impact. Vet Res. 2023;54(1):8. 10.1186/s13567-023-01136-2. 36726112 10.1186/s13567-023-01136-2 PMC9893591

[14] Allan GM, McNeilly F, Walker IW, Young JA, Fee S, Douglas AJ, Adair BM. Serological evidence for Pneumovirus infections in pigs. Vet Rec. 1998;142(1):8–12. 10.1136/vr.142.1.8. 9460216 10.1136/vr.142.1.8

[15] Martín-Valls GE, Li Y, Díaz I, Cano E, Sosa-Portugal S, Mateu E. Diversity of respiratory viruses present in nasal swabs under influenza suspicion in respiratory disease cases of weaned pigs. Front Vet Sci. 2022;9:1014475. 10.3389/fvets.2022.1014475. 36337208 10.3389/fvets.2022.1014475 PMC9627340

[16] Graaf-Rau A, Hennig C, Lillie-Jaschniski K, Koechling M, Stadler J, Boehmer J, Ripp U, Pohlmann A, Schwarz BA, Beer M, Harder T. Emergence of swine influenza A virus, Porcine respirovirus 1 and swine orthopneumovirus in Porcine respiratory disease in Germany. Emerg Microbes Infect. 2023;12(2):2239938. 10.1080/22221751.2023.2239938. 37470510 10.1080/22221751.2023.2239938 PMC10402848

[17] Park J, Kim HR, Lee EB, Lee SK, Kim WI, Lyoo YS, Park CK, Ku BK, Jeoung HY, Lee KK, Park SC. First detection and genetic characterization of swine orthopneumovirus from domestic pig farms in the Republic of Korea. Viruses. 2023;15(12):2371. 10.3390/v15122371. 38140612 10.3390/v15122371 PMC10747143

[18] Richard CA, Hervet C, Ménard D, Gutsche I, Normand V, Renois F, Meurens F, Eléouët JF. First demonstration of the circulation of a Pneumovirus in French pigs by detection of anti-swine orthopneumovirus nucleoprotein antibodies. Vet Res. 2018;49(1):118. 10.1186/s13567-018-0615-x. 30518406 10.1186/s13567-018-0615-x PMC6280484

[19] Core Team R. R: A Language and environment for statistical computing. R Foundation for Statistical Computing; 2024.

[20] Snoeck CJ, Zohari S. Detection and discovery of coronaviruses in wild bird populations, Coronaviruses: Methods and Protocols, 2020, 2203, Humana, New York, NY, USA, 41–53. 10.1007/978-1-0716-0900-2 10.1007/978-1-0716-0900-2_3 32833202

[21] Vigerust DJ, Shepherd VL. Virus glycosylation: role in virulence and immune interactions. Trends Microbiol. 2007;15(5):211–8. 10.1016/j.tim.2007.03.003. 17398101 10.1016/j.tim.2007.03.003 PMC7127133

[22] Owji H, Nezafat N, Negahdaripour M, Hajiebrahimi A, Ghasemi Y. A comprehensive review of signal peptides: Structure, roles, and applications. Eur J Cell Biol. 2018;97(6):422–41. 10.1016/j.ejcb.2018.06.003. 29958716 10.1016/j.ejcb.2018.06.003

[23] Mas V, Nair H, Campbell H, Melero JA, Williams TC. Antigenic and sequence variability of the human respiratory syncytial virus F glycoprotein compared to related viruses in a comprehensive dataset. Vaccine. 2018;36(45):6660–73. 30292456 10.1016/j.vaccine.2018.09.056 PMC6203811

[24] Felsenstein J. Confidence limits on phylogenies: an approach using the bootstrap. Evolution. 1985;39(4):783–91. 10.1111/j.1558-5646.1985.tb00420.x. 28561359 10.1111/j.1558-5646.1985.tb00420.x

[25] Kumar S, Stecher G, Tamura K. MEGA7: molecular evolutionary genetics analysis version 7.0 for bigger datasets. Mol Biol Evol. 2016;33(7):1870–4. 10.1093/molbev/msw054. 27004904 10.1093/molbev/msw054 PMC8210823

[26] Ståhl K, Andersson E, Andersson M, Axén C, Bonnevie A, Bujila I, Chenais E, Dahlquist M, Davidsson L, Dryselius R, Eriksson H, Ernholm L, Fasth C, Grant M, Gröndahl G, Hallgren G, Hansen A, Hjertqvist M, Holmberg M, Hultén CC, Hällbom H, Höök H, Jakobsson K, Jansson D, Jinnerot T, Wensman JJ, Jonsson J, Lindsjö K, Kjellsdotter S, König U, Lahti E, Larsdotter E, Latorre-Margalef N, Lindblad M, Lundén A, Nilsson A, Nilsson OK, Nöremark M, Omazic A, Ordell A, Persson Y, Pettersson E, Ro-Driguez Ewerlöf I, Rosendal T, Sjölund M, Sundqvist L, Söderlund R, Thelander MN, Troell K, Uhlhorn H, Wallensten A, Widgren S, Wikström C, Windahl U, Young B, Yousef N, Zohari S, Ågren E, Ågren E, Gustafsson W. Surveillance of infectious disease in animals and humans in Sweden 2022. -2022-web-2025-04-10.pdf. Assessed 5 Jan 2025. https://www.sva.se/media/ptxlmbkl/surveillance

[27] Carlsson U, Wallgren P, Renström LH, Lindberg A, Eriksson H, Thorén P, Eliasson-Selling L, Lundeheim N, Nörregard E, Thörn C, Elvander M. Emergence of Porcine reproductive and respiratory syndrome in sweden: detection, response and eradication. Transbound Emerg Dis. 2009;56(4):121–31. 10.1111/j.1865-1682.2008.01065.x. 19245667 10.1111/j.1865-1682.2008.01065.x

[28] Atanasova K, Van Gucht S, Barbé F, Lefebvre DJ, Chiers K, Van Reeth K. Lung cell tropism and inflammatory Cytokine-Profile of Porcine respiratory coronavirus infection. Open Vet J. 2008;2:1–10.

[29] SVA. Vaccination av grisar i Sverige. https://www.sva.se/djurhaelsa/djurslag-a-oe/produktionsdjur/gris/smittskydd-foer-gris/vaccination-av-grisar-i-sverige/. Accessed 31 Mar 2025.

[30] Easton AJ, Domachowske JB, Rosenberg HF. Animal pneumoviruses: molecular genetics and pathogenesis. Clin Microbiol Rev. 2004;17(2):390–412. 10.1128/CMR.17.2.390-412.2004. 15084507 10.1128/CMR.17.2.390-412.2004 PMC387412

[31] Borchers AT, Chang C, Gershwin ME, Gershwin LJ. Respiratory syncytial virus–a comprehensive review. Clin Rev Allergy Immunol. 2013;45(3):331–79. 10.1007/s12016-013-8368-9. 23575961 10.1007/s12016-013-8368-9 PMC7090643

[32] Kutter JS, de Meulder D, Bestebroer TM, Mulders A, Fouchier RAM, Herfst S. Comparison of three air samplers for the collection of four nebulized respiratory viruses - Collection of respiratory viruses from air. Indoor Air. 2021;31(6):1874–85. 10.1111/ina.12875. 34124803 10.1111/ina.12875 PMC8530848

[33] Miao Q, Ma Y, Wang Q, Pan J, Zhang Y, Jin W, Yao Y, Su Y, Huang Y, Wang M, Li B, Li H, Zhou C, Li C, Ye M, Xu X, Li Y, Hu B. Microbiological diagnostic performance of metagenomic Next-generation sequencing when applied to clinical practice. Clin Infect Dis. 2018;67(suppl2):S231–40. 10.1093/cid/ciy693. 30423048 10.1093/cid/ciy693

[34] Li N, Cai Q, Miao Q, Song Z, Fang Y, Hu B. High-Throughput metagenomics for identification of pathogens in the clinical settings. Small Methods. 2021;5(1):2000792. 10.1002/smtd.202000792. 33614906 10.1002/smtd.202000792 PMC7883231

