# Supplementary material for: The first detection of swine orthopneumovirus in a pig farm in Sweden: a case report
Source: Porcine Health Manag. 2025 Nov 5;11:56. doi: 10.1186/s40813-025-00473-2 (PMC12590822; doi:10.1186/s40813-025-00473-2)
Supplement: Supplementary file 1 — Supplementary Material 1 [file 40813_2025_473_MOESM1_ESM.docx]

**Nucleic acid extraction**

The RNA extraction was performed using the IndiMag Pathogen Kit w/o plastics (384) (INDICAL Bioscience, Leipzig, Germany). Two hundred μl of sample and 20 μl of proteinase K were manually transferred to a deep 96-well plate, followed by a 500 μl lysate mix, prepared according to the manufacturer's instructions. The RNA extraction was conducted by the Maelstrom-9600 (TANBead, Taoyuan City, Taiwan) extraction robot.

**RT-qPCR**

The SOV RT-qPCR reactions were performed using the AgPath-ID One-Step RT-PCR Reagents kit (Applied Biosystems, Foster City, CA, USA). A final volume of 13 μl PCR master mix and 2 μl extracted RNA template was used for each reaction. Reactions were run on the CFX96 real-time system (BioRad, Hercules, CA, USA) according to the thermo profile AgPath protocol: 45 ℃, 10 minutes (min); 95 ℃, 10 min; and 47 cycles each of 95 ℃, 15 seconds (s); 60 ℃, 45 s. The fluorescence data were collected at the end of each 60°C steps using the FAM filters, and reactions obtaining a CT-value of less than 40 were considered positive.

**Whole genome sequencing**

According to the manufacturer's instructions, library construction was performed using the NEXTERA-XT kit (Illumina Inc. San Diego, CA, USA). The Agilent 2100 Bioanalyzer (Agilent Technologies. Santa Clara, CA, USA) was used to assess the quality of the obtained libraries. The libraries were sequenced on a MiSeq Instrument (Illumina Inc. San Diego, CA, USA) available at the Department of Microbiology, Swedish Veterinary Agency, Uppsala, Sweden, using a Miseq Reagent Kit v3 in a 600-cycle paired-end run. Quality analysis, filtering, and de novo assembly of the raw reads were performed by CLC genomics workbench 23.0.1 (CLC bio, Aarhus, Denmark). Genome annotation was done by using CLC genomics workbench 23.0.1.
